# Prognostic significance of tumor-infiltrating CD8^+^ and FOXP3^+^ lymphocytes in residual tumors and alterations in these parameters after neoadjuvant chemotherapy in triple-negative breast cancer: a retrospective multicenter study

**DOI:** 10.1186/s13058-015-0632-x

**Published:** 2015-09-04

**Authors:** Minoru Miyashita, Hironobu Sasano, Kentaro Tamaki, Hisashi Hirakawa, Yayoi Takahashi, Saki Nakagawa, Gou Watanabe, Hiroshi Tada, Akihiko Suzuki, Noriaki Ohuchi, Takanori Ishida

**Affiliations:** Department of Surgical Oncology, Tohoku University Graduate School of Medicine, 1-1 Seiryo-machi, Aoba-ku, Sendai, 980-8574 Japan; Department of Pathology, Tohoku University Hospital, 1-1 Seiryo-machi, Aoba-ku, Sendai, 980-8574 Japan; Department of Breast Surgery, Nahanishi Clinic, 2-1-9 Akamine, Naha, 901-0154 Japan; Department of Breast Surgery, Tohoku Kosai Hospital, 2-3-11 Kokubuncho, Aoba-ku, Sendai, 980-0803 Japan

## Abstract

**Introduction:**

The status of tumor-infiltrating lymphocytes (TILs) has been recently proposed to predict clinical outcome of patients with breast cancer. We therefore studied the prognostic significance of CD8^+^ TILs and FOXP3^+^ TILs in residual tumors after neoadjuvant chemotherapy (NAC) and the alterations in these parameters before and after NAC in patients with triple-negative breast cancer (TNBC).

**Methods:**

One hundred thirty-one TNBC patients who received NAC at three institutions were examined. CD8^+^ TIL and FOXP3^+^ TIL in residual tumors and biopsy specimens were evaluated by double-staining immunohistochemistry. The CD8^+^ TIL and FOXP3^+^ TIL status of the residual tumors was assessed, and the rates of their changes before and after NAC were calculated.

**Results:**

TNBC patients with high CD8^+^ TIL levels or a high CD8/FOXP3 ratio in residual tumors had significantly better recurrence-free survival (RFS) and breast cancer-specific survival (BCSS) than patients with low values of these parameters. In multivariate analyses, CD8^+^ TIL exhibited strong prognostic significance for RFS, with a hazard ratio (HR) of 3.09 (95 % confidence interval (CI) 1.537–6.614, *P*=0.0013). The CD8/FOXP3 ratio was also significantly correlated with RFS (HR=2.07, 95 % CI 1.029–4.436, *P*=0.0412). TNBC with larger residual tumor size and positive lymph node status, which are known prognostic factors, was independently associated with worse RFS (*P*=0.0064 and *P*=0.0015, respectively). High CD8^+^ TIL levels were a markedly powerful indicator of improved BCSS, with an HR of 3.59 (95 % CI 1.499–9.581, *P*=0.0036). Nodal status was also associated with BCSS (*P*=0.0024). TNBC with a high rate of CD8^+^ TIL changes was associated with significantly better RFS compared with the low group (*P*=0.011). Higher rates of changes in the CD8/FOXP3 ratio were significantly correlated with both better RFS and BCSS compared with lower rates (*P*=0.011 and *P*=0.023, respectively).

**Conclusions:**

This is the first study to demonstrate that high CD8^+^ TIL and a high CD8/FOXP3 ratio in residual tumors and increment of these parameters following NAC and accurately predict improved prognosis in TNBC patients with non-pathological complete response following NAC. These parameters could serve as a surrogate one for adjuvant treatment in patients with residual disease in the neoadjuvant setting.

**Electronic supplementary material:**

The online version of this article (doi:10.1186/s13058-015-0632-x) contains supplementary material, which is available to authorized users.

## Introduction

Triple-negative breast cancer (TNBC) comprises 10 % to 20 % of breast cancers and is characterized by a lack of estrogen receptor (ER), progesterone receptor (PgR), and human epidermal growth factor receptor-2 (HER2) expression. TNBCs are usually composed of biologically aggressive and histologically high-grade tumors and tend to relapse within 3 years of diagnosis with the worst clinical outcome at this juncture [1–3]. Previous clinical trials evaluating the efficacy of chemotherapy in the neoadjuvant setting revealed that TNBCs are more sensitive to chemotherapy and have higher rates of pathological complete response (pCR) than other breast cancer subtypes [4, 5]. In contrast to patients with pCR and good clinical outcome, TNBCs with non-pCR have been reported to be associated with a markedly worse prognosis [4]. Nevertheless, some patients with residual triple-negative tumors following neoadjuvant chemotherapy (NAC) are also known to achieve a relatively long-term survival without recurrence. Therefore, novel biological markers in residual tumors that can predict the survival of non-pCR patients and identify those who should receive new investigational therapeutic agents are required to further improve the patients with TNBC.

Tumor-infiltrating lymphocytes (TILs) have been reported to be associated with clinical outcome of the patients in a number of different malignant tumors, including breast cancer [6–8]. TILs have been reported to differ among breast cancer subtypes and are considered a reliable marker of the efficacy of chemotherapy and trastuzumab in the TNBC and HER2-enriched subtypes [9–12]. Dieci et al. recently reported that the presence of TILs in residual disease following NAC was associated with improved clinical outcome in TNBCs [13]. The results of this study could certainly have clinical relevance for selecting the patients at risk of relapse in TNBCs, but subclassification of the lymphocytes constituting the tumor-associated immune system was not performed in their report. Subclassification of TILs is pivotal; for instance, cytotoxic T cells (CD8^+^ T cells) have been reported to be associated with improved clinical outcomes in patients with breast cancer [14–16], but other studies could not confirm this association [17]. In addition, regulatory T cells defined as forkhead box protein 3 (FOXP3)^+^ T cells play a pivotal role in suppressing anti-tumor immunity [18, 19]. However, it is also true that the prognostic roles of FOXP3 remain controversial; for instance, breast cancers with FOXP3^+^ TIL have been reported to be less sensitive to cytotoxic chemotherapy and have a worse prognosis [17, 20], but others reported that those with FOXP3^+^ TIL have a better prognosis [21, 22].

The results of recent preclinical studies revealed that cytotoxic agents could possibly exert their antitumor activities by inducing an immune response against tumor cells [23, 24]. In a small breast cancer series of 21 patients with residual disease following NAC, chemotherapy-induced lymphocytes in the tumor bed were observed in seven patients [25]. The study of TNBCs by Dieci et al. above also reported that TILs were lower in pre- than post-chemotherapy specimens in 18 of 19 patients [13]. Ladoire et al. analyzed the changes in subclassified TILs, focusing upon CD8 and FOXP3 status in a breast cancer series including 19 cases of ER-negative disease, and reported that the ratio of CD8/FOXP3 following chemotherapy may correlate with improved prognosis [26]. However, more than half of the NAC regimens administered to patients in previous studies were anthracycline-based regimens, whereas the current standard is anthracycline- and taxane-based regimens. In patients with TNBC, the association of CD8^+^ TIL and FOXP3^+^ TIL status in residual tumors and changes induced by anthracycline- and taxane-based chemotherapy with prognosis has therefore remained unknown.

Here, in this study, we evaluated the prognostic significance of CD8^+^ TIL and FOXP3^+^ TIL in residual tumors following NAC comprising anthracyclines and taxanes and the changes of CD8^+^ TIL and FOXP3^+^ TIL before and after the therapy in a relatively larger TNBC cohort than those of previous studies. The aim of our study was to identify a reliable marker for more appropriate selection of high-risk patients eligible for more aggressive therapeutic agents, including novel investigational ones in development in patients with TNBC.

## Methods

### Cohort and sample selection

In this retrospective study, we used data for 131 consecutive TNBC patients who received NAC and surgery at three institutions (Tohoku University Hospital, Sendai, Japan; Tohoku Kosai Hospital, Sendai, Japan; and Nahanishi Clinic, Okinawa, Japan) between 2007 and 2012. All biopsy specimens prior to NAC and surgical specimens were fixed in 10 % neutral buffered formalin and embedded in paraffin. The three institutional review boards, Ethics Committee of Tohoku University Graduate School of Medicine, Institutional Review Board of Tohoku Kosai Hospital, and Institutional Review Board of Nahanishi Clinic, approved the protocol of this study, which was conducted in accordance with the Declaration of Helsinki. Written informed consent was obtained from all patients.

### Double-staining immunohistochemistry and scoring of CD8 and FOXP3

The formalin-fixed paraffin-embedded specimens were cut into 4-μm-thick sections and placed on glue-coated glass slides for immunohistochemistry (IHC). Briefly, the specimens were deparaffinized in xylene and hydrated in graded alcohols and distilled water. Endogenous peroxidase activity was blocked with 3 % hydrogen peroxidase for 10 min. FOXP3 antigen retrieval was performed by autoclaving (Tomy SX-500 High-Pressure Steam Sterilizer, Tomy Seiko Co., Ltd., Tokyo, Japan) in citrate buffer (pH 6.0) and heating at 121 °C for 5 min. The samples were then incubated for 30 min at room temperature (RT) in a blocking solution with 1 % rabbit serum (Nichirei Bioscience, Tokyo, Japan). Then, the anti-FOXP3 antibody reaction (clone 236A/E7; Abcam, Tokyo, Japan) was performed at a dilution of 1:100 for 16 h at 4 °C. A secondary antibody reaction was performed by using a biotinylated rabbit anti-mouse antibody (Nichirei Bioscience) for 30 min at RT. Peroxidase-conjugated streptavidin (Nichirei Bioscience) was subsequently used for 30 min at RT, and DAB was used to visualize the binding of the first antibody. Next, CD8 antigen retrieval was performed by autoclaving in citrate buffer (pH 7.0); the sample was then heated at 121 °C for 5 min and incubated for 30 min at RT in a blocking solution of 1 % rabbit serum. The anti-CD8 antibody reaction (clone C8/144B; Nichirei, Tokyo, Japan) was performed at a dilution of 1:40 for 16 h at 4 °C. After the biotin-streptavidin reaction, Vector Blue® was used to visualize the binding of the anti-CD8 antibody (blue), in contrast to FOXP3 (brown) [27].

The assessment of unstained TILs in our study was based on the recommendation by an International TIL Working Group [8]. TILs were evaluated within the stromal compartment close to the invasive tumor, and the percentage of stromal TILs was reported. To evaluate the tumor infiltration of CD8^+^ and FOXP3^+^ lymphocytes, we performed the experiment, whose procedures were as follows: firstly, four non-overlapping stromal fields with abundant TILs within the borders of the invasive tumor on low-power hematoxylin-eosin-stained field (40×) were selected in both full sections of biopsy specimens and the entire tumor bed of surgical specimens for each case. Then, in the same fields of double staining with CD8 and FOXP3 as the above four fields, the number of CD8^+^ or FOXP3^+^ lymphocytes was counted under high-power magnification (400×) [14, 20]. Finally, the mean number of CD8^+^ or FOXP3^+^ lymphocytes per field was counted, and the ratio of CD8^+^ to FOXP3^+^ lymphocytes was calculated for each tumor. One hundred one surgical specimens and 78 biopsy specimens were scored twice by the same pathologist in order to confirm the reproducibility of the CD8 and FOXP3 scoring. The results indicated that high correlation rates were observed by Pearson correlation analysis (r=0.94–0.96). After that, we assessed the interpersonal reproducibility of the scoring by two blinded pathologists, and high correlation coefficients were obtained (r=0.75–0.88).

### Evaluation of ER, PgR, HER2, Ki-67, EGFR, and CK5/6

The ER and PgR statuses were evaluated by immunostaining using monoclonal antibodies (clone 107925 and 102333, respectively; Roche Diagnostics, Basel, Switzerland); nuclear staining of greater than 1 % was considered positive. HER2 status was evaluated by IHC (HercepTest, code K5204; Dako, Glostrup, Denmark) or by fluorescence *in situ* hybridization (FISH) and was calculated as the gene copy ratio of HER2 to CEP17 (the PathVysion HER2 DNA Probe Kit; Abbott, Chicago, IL, USA). HER2 positivity was defined as 3+ receptor overexpression by IHC or as a HER2-to-CEP17 ratio of at least 2.2 by FISH or as both, in accordance with the American Society of Clinical Oncology/College of American Pathologists guidelines [28].

We performed IHC for Ki-67, EGFR, and CK5/6. The Ki-67 labeling index was determined with an anti-MIB-1 monoclonal antibody (code M7240; Dako) by counting 1000 tumor cells in the hot spots [29, 30]. EGFR was interpreted as positive if the membranes of 10 % or more carcinoma cells were stained by using representative monoclonal antibodies (code K1492; Dako). CK5/6 was interpreted as positive if 10 % or more carcinoma cells displayed monoclonal antibody binding (code M7237; Dako) in the cytoplasm [31, 32]. The basal-like type was defined as tumors expressing either EGFR or CK5/6, which are specific basal markers. Two pathologists performed all pathological diagnoses and staining assessments of individual cases.

### Clinical information, pathological response, and survival

Clinical information for individual TNBC cases was obtained from the databases of the three institutions and was reviewed. The pathological therapeutic response was evaluated at the surgically resected tumor after NAC. The surgical specimens were cut into 5-mm slices and processed with hematoxylin-eosin staining. pCR was defined as the absence of all invasive cancer cells and lymph node metastasis, regardless of the presence or absence of noninvasive cancer cells. We set two survival end points: (i) recurrence-free survival (RFS) was defined as the time from surgery until the date of disease recurrence, and (ii) breast cancer-specific survival (BCSS) was defined as the time until death due to breast cancer.

### Statistical analyses

All statistical analyses were performed by using SAS software, JMP Pro 11 (JMP, Tokyo, Japan). The Kaplan-Meier method and the log-rank test were used to compare the RFS and BCSS curves between groups. Hazard ratios (HRs) and 95 % confidence intervals (CIs) of the variables were calculated by using the Cox proportional hazard regression model. A multivariate Cox regression analysis including all of potential variables that were significantly associated with survival in each univariate analysis was performed. We examined the prognostic significances of TIL-related variables as both a continuous and a categorical variable. Associations among variables were evaluated by using Fisher’s exact test or the chi-squared test. The Mann-Whitney *U* and Spearman’s correlation tests were used to compare non-continuous parameters. All tests were two sided, and a *P* value of less than 0.05 was considered significant.

## Results

### Clinicopathological characteristics and their association with CD8^+^ and FOXP3^+^ TIL

Of the 131 patients with TNBC, 110 (84 %) received NAC containing both anthracyclines and taxanes as current standard regimens. Nineteen patients received anthracycline-based regimens for NAC, and the great majority received taxane-based regimens as adjuvant therapy after surgery. Thirty patients (30/131; 22.9 %) were diagnosed as having pCR according to the detailed pathological examination.

**Fig. 1:**
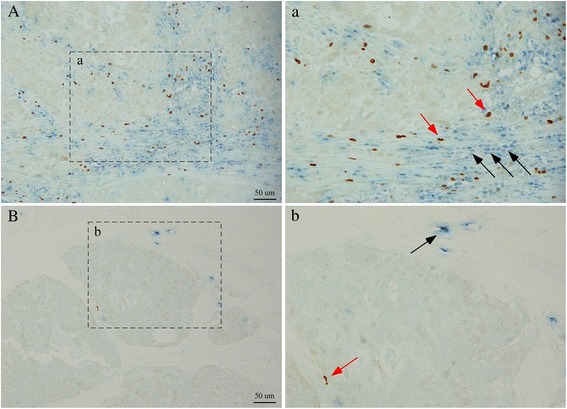
Immunohistochemical double staining of tumor-infiltrating CD8^+^ (*blue*) and FOXP3^+^ (*brown*) lymphocytes. The representative tumor tissue of high (**a**) and low (**b**) infiltration of lymphocytes on surgical specimens after neoadjuvant chemotherapy (*black arrow* points to CD8^+^ lymphocytes, and *red arrow* points to FOXP3^+^ lymphocytes)

Immunohistochemical analyses of CD8, FOXP3, EGFR, CK5/6, and Ki-67 and the CD8^+^/FOXP3^+^ ratio by the double-staining method were subsequently performed in residual tumors from 101 patients with TNBC treated with NAC, and representative images are shown in Fig. 1. The characteristics of the 101 evaluable TNBC patients with respect to CD8 and FOXP3 status and the CD8/FOXP3 ratio are presented in Table 1. A total of 85 of these patients (84 %) received anthracycline- and taxane-based neoadjuvant regimens, and most of the others received anthracycline-based neoadjuvant regimens and taxane-based regimens after surgery. The cutoffs for high or low infiltration were defined as near the median number of infiltrating cells per field as follows: CD8, 100 infiltrating cells per field; FOXP3, 60 infiltrating cells per field; and CD8/FOXP3 ratio, 1.6. There was no difference in CD8^+^ TIL and FOXP3^+^ TIL status and CD8/FOXP3 ratio among the NAC regimens. TNBC patients with small residual tumor size (≤2 cm) after NAC comprised 59/101 (58 %) and had significantly higher CD8^+^ TIL levels than patients with residual tumors larger than 2 cm (*P*=0.005). There was no association between residual tumor size and FOXP3^+^ TIL or the CD8/FOXP3 ratio. Residual tumors with higher histological grades and higher Ki-67 with a cutoff of 50 % near the median value tended to have lower CD8/FOXP3 ratios, but this trend was not significant (*P*=0.068 and 0.056, respectively). Significant positive correlations were detected among CD8^+^ TIL, FOXP3^+^ TIL, and CD8/FOXP3 ratio (CD8^+^ versus FOXP3^+^: *P*=0.0003, CD8^+^ versus CD8/FOXP3 ratio: *P*=0.010, FOXP3^+^ versus CD8/FOXP3 ratio: *P*=0.0001). No correlation of other factors, age, nodal status after NAC, and basal-like type with high or low CD8^+^ TIL, FOXP3^+^ TIL, and CD8/FOXP3 ratio was observed.

**Table 1:** Clinicopathological factors of triple-negative breast tumors with the status of CD8, FOXP3, the ratio of CD8/FOXP3 (*N*=101)

Factors	CD8			FOXP3			CD8/FOXP3		
	High	Low	*P* value	High	Low	*P* value	High	Low	*P* value
Age, years			0.838			0.415			0.682
<50	22	17		22	17		16	23	
>50	33	29		29	33		29	33	
Residual tumor size			0.005*			0.373			0.487
<2.0 cm	39	20		32	27		28	31	
>2.0 cm	16	26		19	23		17	25	
Nodal status after NAC			0.167			0.491			0.094
Negative	35	23		31	27		30	28	
Positive	20	23		20	23		15	28	
Residual cancer burden			0.052			0.908			0.013*
Class I	5	2		3	4		6	1	
Class II	41	27		35	33		32	36	
Class III	9	17		13	13		7	19	
Histological grade			0.602			0.329			0.068
I, II	21	16		15	22		22	17	
III	34	30		36	28		23	39	
Neoadjuvant chemotherapy			0.726			0.319			0.136
Anthracycline and taxane-based	45	40		43	42		39	46	
Anthracycline-based	9	5		8	6		4	10	
Other	1	1		0	2		2	0	
Basal-like type			0.314			0.546			0.417
Basal-like	30	30		32	28		29	31	
Non basal-like	25	16		19	22		16	25	
Ki-67 LI (cutoff: 50)			0.551			0.322			0.056
Low	26	25		23	28		28	23	
High	29	21		28	22		17	33	
CD8 (cutoff: 100)						0.0003*			0.010*
Low				14	32		14	32	
High				37	18		31	24	
FOXP3 (cutoff: 60)			0.0003*						0.0001*
Low	18	32					32	18	
High	37	14					13	38	
CD8/FOXP3 (cutoff: 1.6)			0.010*			0.0001*			
Low	24	32		38	18				
High	31	14		13	32				

*NAC* neoadjuvant chemotherapy, *LI* labeling index

*The *P* value is significant

We also assessed residual cancer burden (RCB) scores on surgical specimens of non-pCR patients by using the Web-based MD Anderson RCB calculator and its relationship with CD8^+^ TIL, FOXP3^+^ TIL, and CD8/FOXP3 ratio [33]. The weak inverse relations were detected between RCB scores and CD8^+^ TIL or CD8/FOXP3 ratio (Additional file 1A, C), but this trend was not observed between RCB scores and FOXP3^+^ TIL (Additional file 1B). From the evaluation of RCB categories, significant correlations were detected between RCB classes and CD8/FOXP3 ratio (*P*=0.013, Table 1).

### Association of CD8^+^ TIL, FOXP3^+^ TIL, and clinicopathological factors with prognosis

**Fig. 2:**
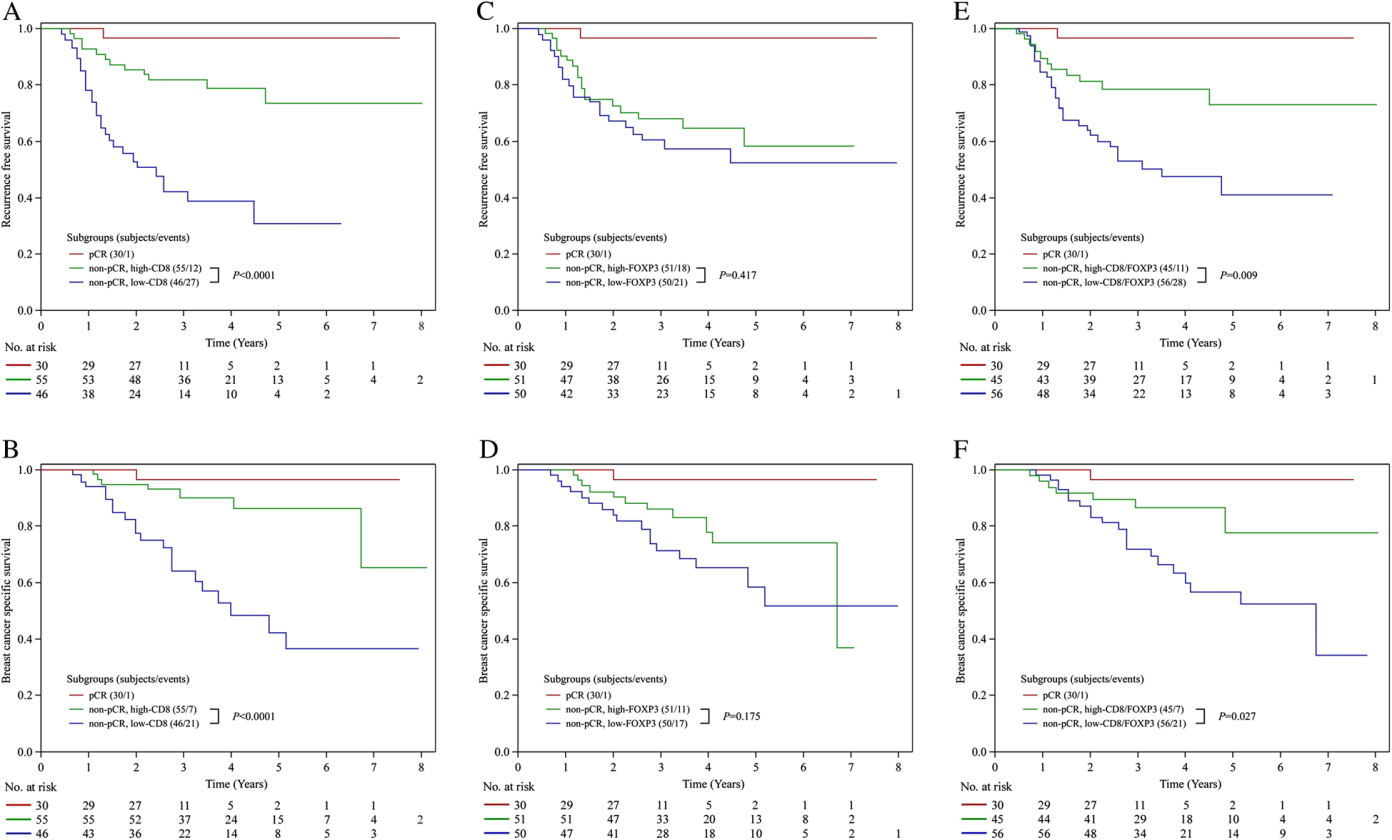
Recurrence-free survival (RFS) and breast cancer-specific survival (BCSS) in patients with different status of CD8^+^ tumor-infiltrating lymphocyte (TIL), FOXP3^+^ TIL, and CD8/FOXP3 ratio. Estimated Kaplan-Meier curves of RFS (**a**) and BCSS (**b**) in patients with high or low CD8^+^ TIL, those of RFS (**c**) and BCSS (**d**) in patients with high or low FOXP3^+^ TIL, and those of RFS (**e**) and BCSS (**f**) in patients with high or low CD8/FOXP3 ratio. *pCR* pathological complete response

The median follow-up time was 3.6 years. TNBCs classified into the high CD8^+^ TIL group for residual tumors had significantly better RFS and BCSS than the low CD8^+^ TIL group as determined by the Kaplan-Meier method and the log-rank test (*P*<0.0001, both end points). The 5-year RFS rates were 73 % for the high CD8^+^ TIL group and 30 % for the low CD8^+^ TIL group, and the 5-year BCSS rates were 86 % for the high CD8^+^ TIL group and 42 % for the low CD8^+^ TIL group (Fig. 2a, b). A higher CD8/FOXP3 ratio was also significantly correlated with both better RFS and BCSS compared with a lower CD8/FOXP3 ratio (*P*=0.009 and *P*=0.027, respectively). The 5-year RFS rates were 72 % and 40 % in patients with high and low CD8/FOXP3 ratios, respectively, and the 5-year BCSS rates were 77 % and 56 % in patients with high and low CD8/FOXP3 ratios, respectively (Fig. 2e, f). By contrast, no association between the status of FOXP3^+^ TIL and RFS or BCSS was observed (*P*=0.417 and *P*=0.175, respectively; Fig. 2c, d).

Patients with 2 cm or smaller residual tumors had a significantly better prognosis than those with residual tumors larger than 2 cm (RFS, *P* <0.0001; BCSS, *P*=0.005; Additional file 2A, B). No residual axillary lymph node disease after NAC was significantly associated with improved RFS and BCSS compared with residual lymph node metastases (*P* <0.0001, both end points; Additional file 2C, D). Although patients with a basal-like type who did not achieve pCR had a worse RFS than those with a non-basal-like type (defined as not expressing EGFR or CK5/6; *P*=0.042), the BCSS curve did not differ between these types (*P*=0.269; Additional file 3A, B). There was no correlation between the two survival end points and Ki-67 status (RFS, *P*=0.349; BCSS, *P*=0.132; Additional file 3C, D) or the other clinicopathological factors: age, histological grade, and type of NAC regimen.

Additionally, we evaluated the status of unstained TILs according to the recommendation by an International TIL Working Group 2014 [8]. The patients classified into the high TIL group with the recommended cutoff of 60 % underwent the significantly better clinical course compared with those with low TILs regarding RFS (*P*=0.046; Additional file 4A). On the other hand, the BCSS curves were not statistically different between high and low TIL groups (Additional file 4B). The shorter follow-up time probably influenced the discordant results between RFS and BCSS.

### Prognostic value of CD8^+^ and FOXP3^+^ TIL on residual tumor in univariate and multivariate analysis

Both univariate and multivariate analyses were performed to determine the association between prognosis and variables, including clinicopathological factors, CD8, FOXP3, the CD8/FOXP3 ratio, unstained TILs, basal-like status, and Ki-67. As reported in Table 2, CD8^+^ TIL, the CD8/FOXP3 ratio, unstained TILs, residual tumor size, nodal status after NAC, and basal-like type were significantly correlated with RFS in univariate analyses. We investigated these variables for their independent association with RFS by using a multivariate Cox regression model. The results did reveal that CD8^+^ TIL had strong prognostic significance for RFS with HRs of 1.01 (95 % CI 1.001–1.011, *P*=0.0249) as a continuous variable (per 1 point increase) and 3.09 (95 % CI 1.537–6.614, *P*=0.0013) as a categorical variable. The CD8/FOXP3 ratio as a categorical variable was also significantly correlated with RFS (HR=2.07, 95 % CI 1.029–4.436, *P*=0.0412), and that was suggestively associated with RFS as a continuous variable (HR=1.14 per 1 point increase, 95 % CI 0.994–1.381, *P*=0.0631). TNBC patients with larger residual tumor size and positive lymph node status, known prognostic factors, independently exhibited worse RFS (HR=2.59, 95 % CI 1.302–5.396, *P*=0.0064; HR=2.97, 95 % CI 1.509–6.178, *P*=0.0015, respectively). With regard to BCSS, CD8^+^ TIL, the CD8/FOXP3 ratio, residual tumor size, and nodal status after NAC were significantly correlated with survival in the univariate analyses (Table 3). No difference in the BCSS end point was detected for basal-like type and non-basal-like type tumors. In multivariate analyses, the results indicated that a high number of CD8^+^ TILs was a markedly powerful indicator of improved BCSS, with HRs of 1.01 (95 % CI 1.001–1.015, *P*=0.0152) as a continuous variable (per 1 point increase) and 3.59 (95 % CI 1.499–9.581, *P*=0.0036) as a categorical variable. Nodal status after NAC was also significantly associated with BCSS (HR=3.76, 95 % CI 1.574–10.02, *P*=0.0024; Table 3).

**Table 2:** Univariate and multivariate analyses of variables associated with RFS in TNBC (*N*=101)

Variables	Univariate analysis			Multivariate analysis		
	HR	95 % CI	*P* value	HR	95 % CI	*P* value
Age (<50 vs. >50)	1.45	0.765–2.727	0.2488			
Residual tumor size (>2.0 cm vs. <2.0 cm)	3.92	2.045–7.902	<0.0001*	2.59	1.302–5.396	0.0064*
Nodal status after NAC (positive vs. negative)	3.84	2.003–7.732	<0.0001*	2.97	1.509–6.178	0.0015*
Grade (III vs. I, II)	1.01	0.352–4.234	0.9903			
Basal-like vs. non basal-like	2.03	1.035–4.267	0.0389*	2.01	0.979–4.262	0.0532
Ki-67 LI (high vs. low)	1.35	0.716–2.562	0.3526			
Unstained TILs (low vs. high)	3.63	1.110–22.34	0.0301*	2.01	0.572–12.71	0.3101
CD8 (low vs. high)	3.92	2.016–8.105	<0.0001*	3.09	1.537–6.614	0.0013*
CD8 (as a continuous variable)^a^	1.01^b^	1.002–1.012	0.0021*	1.01^b^	1.001–1.011	0.0249*
FOXP3 (low vs. high)	1.30	0.689–2.456	0.4199			
FOXP3 (as a continuous variable)^a^	1.00^b^	0.997–1.008	0.3753			
CD8/FOXP3 (low vs. high)	2.43	1.239–5.097	0.0090*	2.07	1.029–4.436	0.0412*
CD8/FOXP3 (as a continuous variable)^a^	1.28^b^	1.085–1.616	0.0011*	1.14^b^	0.994–1.381	0.0631

Multivariate cox regression analyses were performed for all potential variables that were significantly associated with survival in univariate analysis

*RFS* recurrence free survival, *HR* hazard ratio, *CI* confidence interval, *NAC* neoadjuvant chemotherapy, *LI* labeling index, *TIL* tumor-infiltrating lymphocyte

*The *P* value is significant

^a^Analyses were performed by the continuous variables

^b^The HRs of the continuous variables are shown as a unit ratio

**Table 3:** Univariate and multivariate analyses of variables associated with BCSS in TNBC (*N*=101)

Variables	Univariate analysis			Multivariate analysis		
	HR	95 % CI	*P* value	HR	95 % CI	*P* value
Age (<50 vs. 50<)	1.85	0.877–3.944	0.1055			
Residual tumor size (>2.0 cm vs. <2.0 cm)	2.89	1.354–6.520	0.0059*	1.37	0.593–3.332	0.4637
Nodal status after NAC (positive vs. negative)	4.83	2.144–12.29	<0.0001*	3.76	1.574–10.02	0.0024*
Grade (III vs. I, II)	1.45	0.460–2.173	0.4014			
Basal-like vs. non basal-like	1.56	0.721–3.635	0.2634			
Ki-67 LI (high vs. low)	1.78	0.839–3.935	0.1329			
Unstained TILs (low vs. high)	2.38	0.709–14.77	0.1815			
CD8 (low vs. high)	4.75	2.106–12.12	0.0001*	3.59	1.499–9.581	0.0036*
CD8 (as a continuous variable)^a^	1.01^b^	1.004–1.016	0.0007*	1.01^b^	1.001–1.015	0.0152*
FOXP3 (low vs. high)	1.68	0.795–3.699	0.1751			
FOXP3 (as a continuous variable)^a^	1.00^b^	0.998–1.013	0.1412			
CD8/FOXP3 (low vs. high)	2.53	1.128–6.436	0.0233*	1.28	0.526–3.453	0.5932
CD8/FOXP3 (as a continuous variable)^a^	1.25^b^	1.048–1.649	0.0077*	1.11^b^	0.953–1.397	0.2115

Multivariate cox regression analyses were performed for all potential variables that were significantly associated with survival in univariate analysis

*BCSS* breast cancer-specific survival, *HR* hazard ratio, *CI* confidence interval, *NAC* neoadjuvant chemotherapy, *LI* labeling index, *TIL* tumor-infiltrating lymphocyte

*The *P* value is significant

^a^Analyses were performed by the continuous variables

^b^The HRs of the continuous variables are shown as a unit ratio

### Changes of CD8^+^ and FOXP3^+^ TIL between before and after NAC and their association with prognosis

**Fig. 3:**
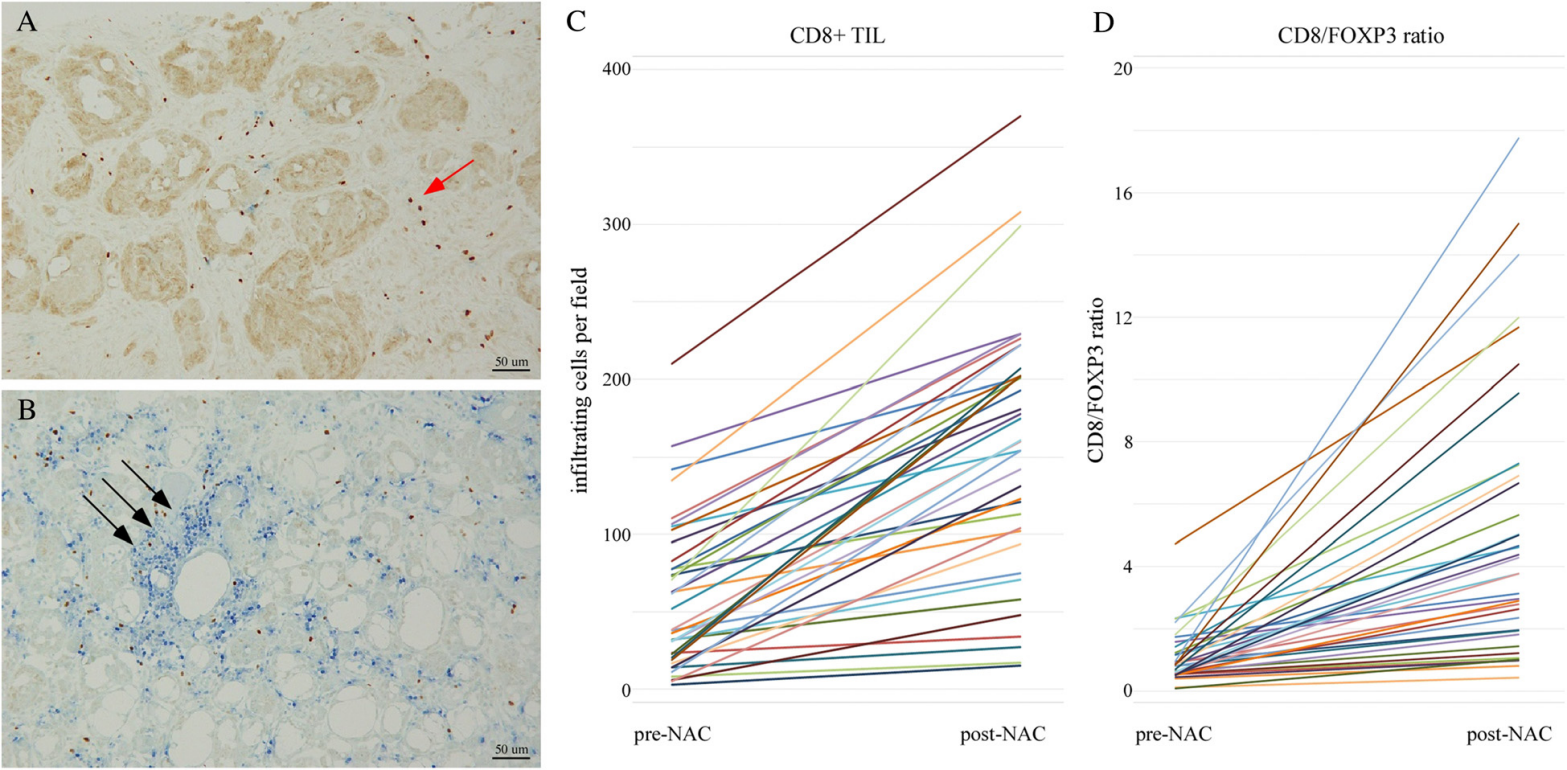
The cases with high change rates of CD8^+^ TIL and CD8/FOXP3 ratio before and after neoadjuvant chemotherapy (NAC). The cutoffs of a high or low rate of changes were defined as near the median ratio as follows: CD8^+^ TIL, 1.4 and CD8/FOXP3 ratio, 1.8. Immunohistochemical images of tumor-infiltrating CD8^+^ (*blue*) and FOXP3^+^ (*brown*) lymphocytes in pretreatment biopsy specimens (**a**) and residual tumors after NAC (**b**) of the same patient (*black arrow* points to CD8^+^ lymphocytes, and *red arrow* points to FOXP3^+^ lymphocytes). **c** Changing levels of CD8^+^ TIL in each case; 9 of 38 cases experienced cancer recurrence, and 7 patients died. **d** Changing levels of CD8/FOXP3 ratio in each case; 8 of 37 cases experienced cancer recurrence, and 5 patients died

**Fig. 4:**
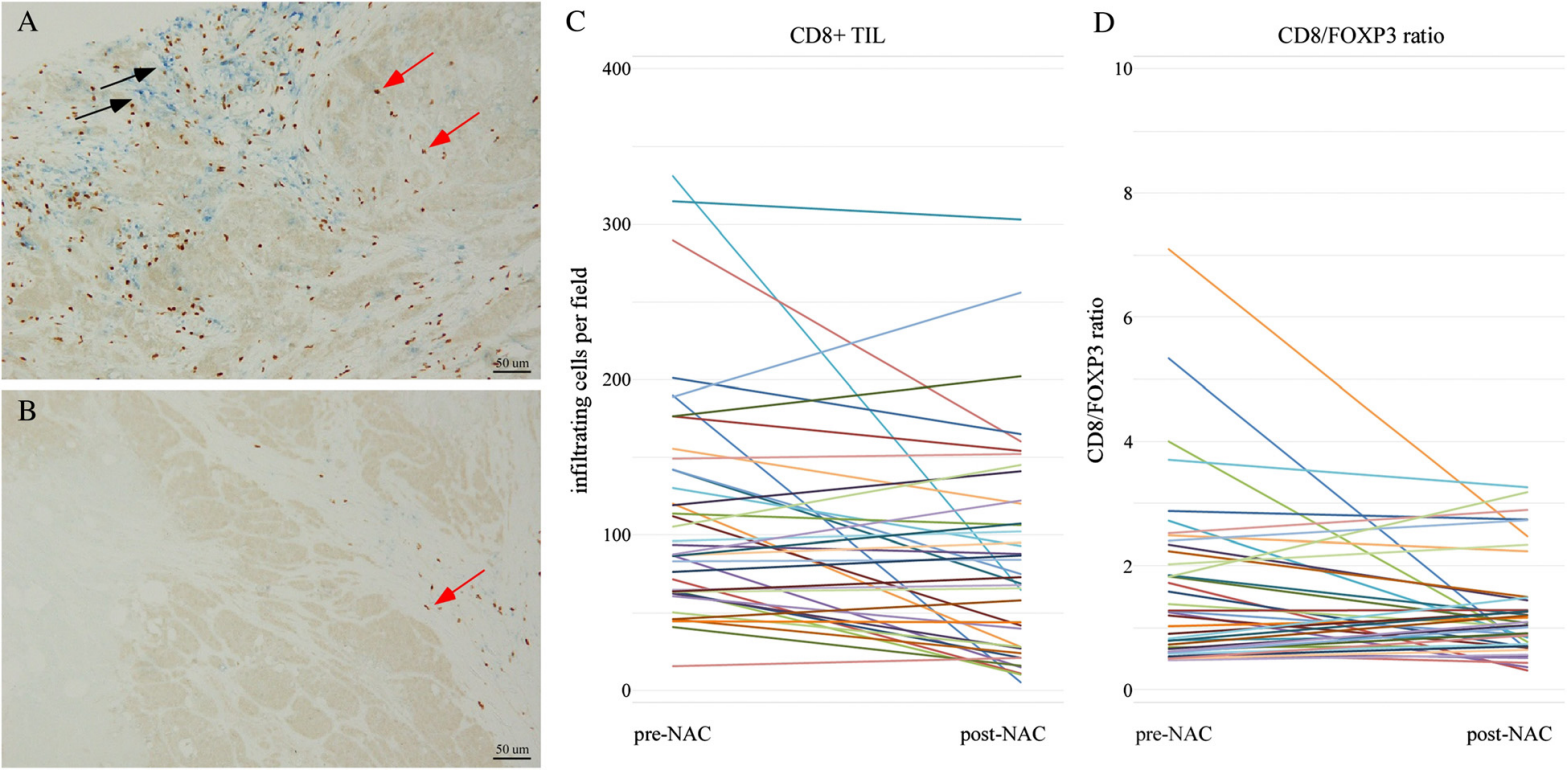
The cases with low change rates of CD8^+^ TIL and CD8/FOXP3 ratio before and after neoadjuvant chemotherapy (NAC). The cutoffs of a high or low rate of changes were defined as near the median ratio as follows: CD8^+^ TIL, 1.4 and CD8/FOXP3 ratio, 1.8. Immunohistochemical images of tumor-infiltrating CD8^+^ (*blue*) and FOXP3^+^ (*brown*) lymphocytes in pretreatment biopsy specimens (**a**) and residual tumors after NAC (**b**) of the same patient (*black arrow* points to CD8^+^ lymphocytes, and *red arrow* points to FOXP3^+^ lymphocytes). **c** Changing levels of CD8^+^ TIL in each case; 20 of 40 cases experienced cancer recurrence and 14 patients died. **d** Changing levels of CD8/FOXP3 ratio in each case; 21 of 41 cases experienced cancer recurrence, and 16 patients died

We investigated the status of CD8^+^ TIL and FOXP3^+^ TIL and the CD8/FOXP3 ratio in the core biopsy specimens and calculated the rates of changes before and after NAC. Pre-treatment biopsy specimens were available for 78 of the 101 included TNBCs. CD8^+^ TIL levels increased after NAC in 54 of 78 patients (69 %), and the change rates varied from 0.03 to 20.08 (the ratio of post-treatment CD8^+^ TIL to pre-treatment CD8^+^ TIL) (Figs. 3 and 4). FOXP3^+^ TIL increased in 35 of 78 patients (45 %), and the change rates varied from 0.02 to 11.00 (the ratio of post-treatment FOXP3^+^ TIL to pre-treatment FOXP3^+^ TIL). In 37 of 78 patients (47 %), the CD8/FOXP3 ratio increased, and the change rates varied from 0.12 to 18.32 (the ratio of post-treatment CD8/FOXP3 ratio to pre-treatment CD8/FOXP3 ratio) (Figs. 3 and 4).

In 38 patients with a high rate of changes in CD8^+^ TILs, nine cases experienced cancer recurrence. Of the 38 patients, 25 cases were classified into the high-change group of CD8/FOXP3 ratio and 13 cases were classified into the low-change group. There were 5/25 (20 %) and 4/13 (31 %) cases with recurrence in the high- and low-change groups of CD8/FOXP3 ratio, respectively. The recurrence rates of the two groups were not statistically different (*P*=0.4645). Twenty of 40 patients with a low rate of changes in CD8^+^ TILs experienced cancer recurrence. In 28 patients with a low rate of changes in both CD8^+^ TILs and CD8/FOXP3 ratio, 17/28 (61 %) cases underwent recurrence which was significantly higher than that of patients with a high rate of changes in CD8/FOXP3 ratio, 3/12 (25 %) (*P*=0.0352).

**Fig. 5:**
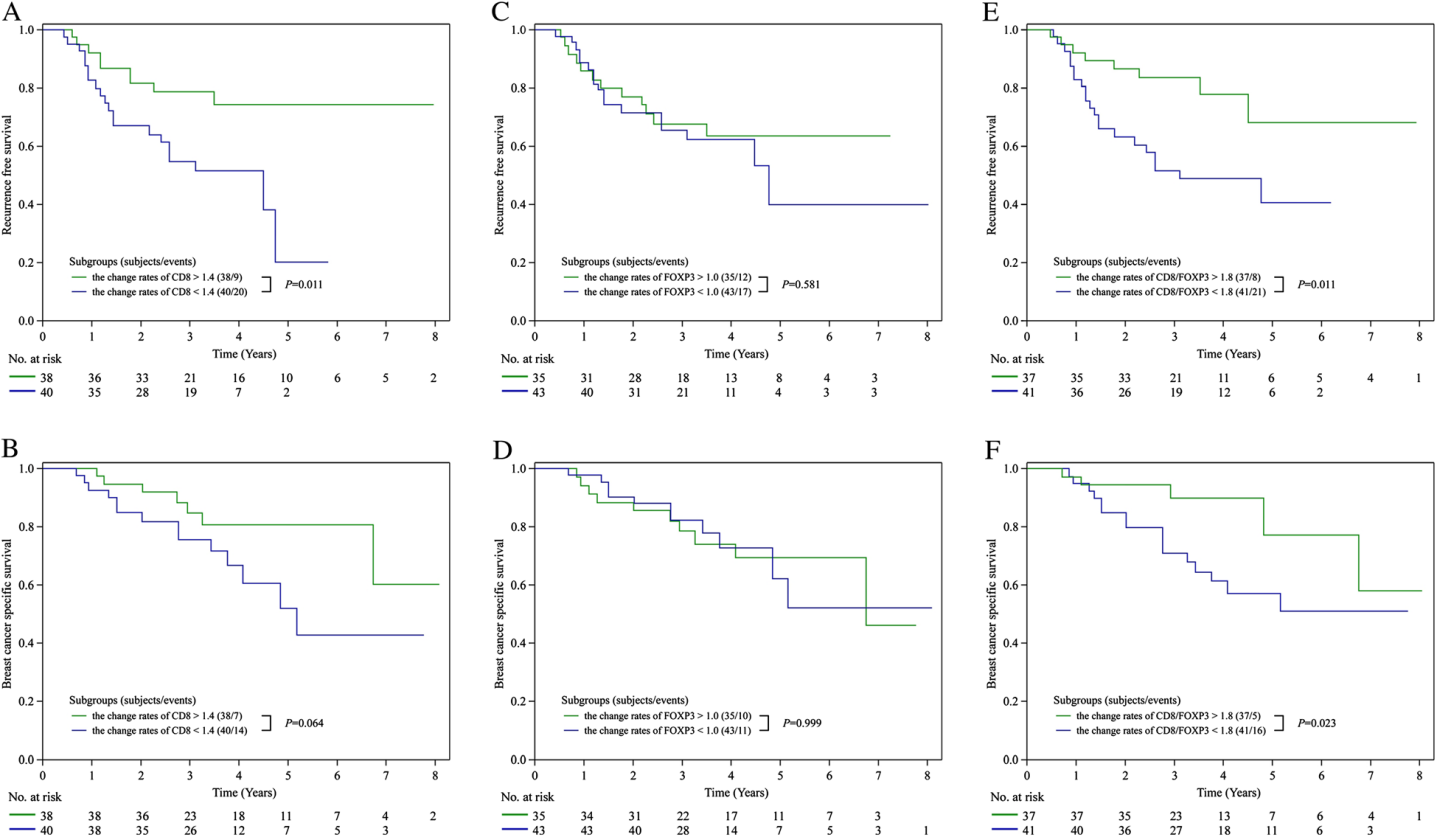
Recurrence-free survival (RFS) and breast cancer-specific survival (BCSS) in patients with different change rates of CD8^+^ tumor-infiltrating lymphocyte (TIL), FOXP3^+^ TIL, and CD8/FOXP3 ratio. Estimated Kaplan-Meier curves of RFS (**a**) and BCSS (**b**) in patients with high or low change rates of CD8^+^ TIL before and after neoadjuvant chemotherapy, those of RFS (**c**) and BCSS (**d**) in patients with high or low change rates of FOXP3^+^ TIL, and those of RFS (**e**) and BCSS (**f**) in patients with high or low change rates of CD8/FOXP3 ratio

We investigated survival with regard to the different change rates of CD8^+^ TIL and FOXP3^+^ TIL status and the CD8/FOXP3 ratio by using the Kaplan-Meier method and log-rank test. The cutoffs of high or low rate of changes were defined as near the median ratio of post-treatment value to pre-treatment value in our TNBC cohort as follows: CD8, 1.4; FOXP3, 1.0; and CD8/FOXP3, 1.8. TNBCs classified into the high rate of change in CD8^+^ TIL group had a significantly better RFS than those with a low rate of change (*P*=0.011), and BCSS was suggestively better in the high rate of change in the CD8^+^ TIL group compared with the low-rate group (*P*=0.064). The 5-year RFS rates were 74 % for the high rate of change in CD8^+^ TIL group and 20 % for the low-rate group, and the 5-year BCSS rates were 81 % for the high rate of change in CD8^+^ TIL group and 52 % for the low-rate group (Fig. 5a, b). The patients with higher change of the CD8/FOXP3 ratio had significantly better RFS and BCSS compared with those not (*P*=0.011 and *P*=0.023, respectively). The 5-year RFS rates were 68 % and 41 % in patients with high and low rates of changes in the CD8/FOXP3 ratio, and the 5-year BCSS rates were 78 % and 58 % in patients with high and low rates of changes in the CD8/FOXP3 ratio, respectively (Fig. 5e, f). However, no significant correlation was detected between the degrees of FOXP3^+^ TIL change and RFS or BCSS (*P*=0.581 and *P*=0.999, respectively; Fig. 5c, d).

## Discussion

Our study is the first to evaluate CD8^+^ TIL, FOXP3^+^ TIL, and the CD8/FOXP3 ratio in residual tumors and changes in these parameters after chemotherapy in more than 100 patients with TNBC. The results indicated that a high CD8^+^ TIL and the high CD8/FOXP3 ratio in residual tumors and increases in these parameters after NAC compared with pretreatment status in biopsy samples can accurately predict improved RFS and BCSS in TNBC patients with non-pCR following NAC.

A recent meta-analysis of studies exploring the prognostic value of TILs in patients with TNBC revealed that high TIL levels were significantly associated with better survival outcome in TNBCs, and the authors concluded that TIL status should be considered a strong prognostic factor in this subtype [10]. Dieci et al. also reported the importance of TIL in residual disease after NAC in the largest cohort of 278 patients with TNBC, and their results have had a large impact in both preclinical and clinical practice [13]. Focusing on the subsets of TIL, the tumor-related immune system has two paradoxically functional components: cytotoxic CD8^+^ T cells and regulatory FOXP3^+^ T cells. Cytotoxic T cells recognize foreign antigens on tumor cells through specific interactions with T-cell receptors, which lead to tumor cell death by inducing the release of proteins such as perforin and granzyme from activated T cells [34–36]. By contrast, FOXP3^+^ TILs, which diminish the immune response to self-antigens, have a critical role in suppressing anti-tumor immunity [18, 19]. Therefore, the evaluation of these two major components of the tumor-related immune system is required for the assessment of chemotherapeutic efficacy in patients with breast cancer.

Based on the limited data on the detailed subclassification of TILs as CD8^+^ TIL or FOXP3^+^ TIL or not in TNBCs, studies evaluating the association of CD8^+^ TIL or FOXP3^+^ TIL status with clinical outcome have reported conflicting findings [14–17, 20–22, 26, 27]. In the above-mentioned meta-analysis, the absence of CD8^+^ TIL was reported to be associated with worse disease-free survival and overall survival (HR=0.24, 95 % CI 0.12–0.45, *P* <0.0001, HR=0.58, 95 % CI 0.52–0.65, *P* <0.0001, respectively), and the absence of FOXP3^+^ TIL^+^ also correlated with worse disease-free survival and overall survival (HR=0.44, 95 % CI 0.27–0.72, *P*=0.001, HR=0.76, 95 % CI 0.60–0.96, *P*=0.019, respectively) [10]. This pooled estimation focusing on CD8^+^ TIL and FOXP3 TIL^+^ was considered valuable, but the results should be interpreted carefully because studies of the value of TIL in the neoadjuvant setting were excluded from this meta-analysis. Therefore, our present retrospective study of the status of CD8^+^ TIL and FOXP3^+^ TIL in residual tumors after NAC could provide results directly connected to present clinical practices, in which NAC is the major treatment mode for patients with TNBC. The positive correlation between high CD8^+^ TIL in residual tumors after NAC and improved prognosis identified in this study is consistent with several previous studies investigating the value of CD8^+^ TILs in the adjuvant setting for TNBCs [14–16]. In addition, the importance of the CD8/FOXP3 ratio following chemotherapy in our TNBC cohorts supports the results of a unique small study that suggested that the ratio of CD8/FOXP3 after chemotherapy is correlated with prognosis [26].

The additional prognostic value of stained TILs, CD8^+^ TILs, FOXP3^+^ TILs, and CD8/FOXP3 ratio should be discussed because there have been several studies which find the significance of unstained TILs in patients with TNBC [8, 10–13]. In our cohort, the positive correlation was observed between the status of unstained TILs and CD8^+^ TILs (R=0.432, *P* <0.001). This is thought to be the statistical reason that unstained TILs were not significant in our multivariate analysis; on the contrary, CD8^+^ TILs were significant. Based on the results, staining CD8 enabled us to identify the patients with poor prognosis more effectively than the unstained TILs which could be more available than stained TILs in clinical practices.

The results of preclinical studies indicate that the demise of immunogenic cells induced by cytotoxic agents could enable the cross-presentation of antigens, dendritic cell activation, and the induction of tumor-specific cytotoxic T cells labeled by CD8, a major component of the tumor-related immune system [23, 24]. To further clarify the dynamic change in tumor infiltration by lymphocytes in patient samples, the changes in CD8^+^ TIL and FOXP3^+^ TIL caused by chemotherapy compared with baseline in biopsy specimens were determined for the first time in this study. We focused on the changes in CD8^+^ TIL, FOXP3^+^ TIL, and the CD8/FOXP3 ratio induced by anthracycline- and taxane-based NAC in a cohort of 78 patients with TNBC. Our study demonstrated that increases in CD8^+^ TIL and the CD8/FOXP3 ratio were significantly associated with improved clinical outcomes. These results suggest that activation of the activated tumor-related immune system by cytotoxic agents might affect the micro-metastatic tumor cells in the bone marrow or blood vessels or other parts of the body, a hypothesis that certainly merits further investigation.

The present study indicates that the post-chemotherapy status of CD8^+^ TIL and the CD8/FOXP3 ratio and the changes in these parameters caused by chemotherapy could be used to identify a subgroup of patients eligible for clinical trials of investigational drugs. Recently, a novel approach targeting programmed death 1 (PD-1) and the PD-1 ligand (PD-L1) pathway has been investigated in human patients with breast cancer [37, 38]. PD-1 is a member of the CD28/cytotoxic T-lymphocyte antigen 4 (CTLA-4) family of co-stimulatory receptors and is a negative regulator of T-cell lymphocytes [39, 40]. The PD-1 ligand PD-L1 is expressed on various tumor cells, including breast cancer cells [38]. Early-phase clinical trials using anti-PD-1 and PD-L1 antibodies have demonstrated favorable objective responses in patients with non-small cell lung cancer, malignant melanoma, and renal cell carcinoma [41–43]. For breast cancer, a phase II trial of an anti-PD-1 antibody will soon be initiated on the basis of the results of a phase I trial that demonstrated durable efficacy and acceptable safety in patients with heavily treated TNBC (the results of the study were presented at the 37th San Antonio Breast Cancer Symposium but have not yet been published). However, the association between the presence of CD8^+^ TIL, FOXP3^+^ TIL, PD-1, PD-L1, and the therapeutic efficacy of an anti-PD-1 antibody or PD-L1 antibody has not been examined. Further studies may be required for patient stratification for the development of these drugs.

As a potential limitation of our study, the status of TILs on biopsy specimens may not reflect that of the entire tumor before chemotherapy because of the tumor heterogeneity. By carefully estimating all pieces (four or more) of biopsy specimens, we minimized the limitation which was common among the studies with neoadjuvant settings. Further studies are needed to validate the results of the present study.

## Conclusions

This is the first study to demonstrate that high CD8^+^ TIL levels and CD8/FOXP3 ratio in residual tumors could accurately predict the better clinical outcome in TNBC patients with non-pCR following NAC and that the changes of these parameters in breast cancer tissues following NAC were also significantly associated with eventual clinical outcome in TNBCs. These parameters could be a surrogate for adjuvant treatment in patients with residual disease in the neoadjuvant setting.

## Additional files

Additional file 1:
**The correlation diagrams of CD8** ^**+**^ **TIL, FOXP3** ^**+**^ **TIL, and CD8/FOXP3 ratio with residual cancer burden (RCB) score of each tumor.** The weak inverse relations were detected between RCB scores and CD8^+^ TIL (**A**) or CD8/FOXP3 ratio (**C**), but this trend was not observed between RCB scores and FOXP3^+^ TIL (**B**). *FOXP3* forkhead box protein 3, *TIL* tumor-infiltrating lymphocyte. (JPEG 599 kb) Additional file 2:
**Recurrence-free survival (RFS) and breast cancer-specific survival (BCSS) in patients with different residual tumor size and lymph node status.** Estimated Kaplan-Meier curves of RFS (**A**) and BCSS (**B**) in patients with residual tumors of more than 2 cm or not more than 2 cm and those of RFS (**C**) and BCSS (**D**) in patients with metastatic lymph node-positive or -negative tumors. (JPEG 610 kb) Additional file 3:
**Recurrence-free survival (RFS) and breast cancer-specific survival (BCSS) in patients with different status of basal-like feature and Ki-67 labeling index (LI).** Estimated Kaplan-Meier curves of RFS (**A**) and BCSS (**B**) in patients with basal-like type or non-basal-like type and those of RFS (**C**) and BCSS (**D**) in patients with high or low Ki-67 LI. (JPEG 590 kb) Additional file 4:
**Recurrence-free survival (RFS) and breast cancer-specific survival (BCSS) in patients with different status of tumor-infiltrating lymphocytes (TILs).** Estimated Kaplan-Meier curves of RFS (**A**) and BCSS (**B**) in patients with high or low TILs. (JPEG 550 kb)

## Abbreviations

BCSS: Breast cancer-specific survival
CI: Confidence interval
ER: Estrogen receptor
FISH: Fluorescence *in situ* hybridization
FOXP3: Forkhead box protein 3
HER2: Human epidermal growth factor receptor-2
HR: Hazard ratio
IHC: Immunohistochemistry
NAC: Neoadjuvant chemotherapy
pCR: Pathological complete response
PD-1: Programmed death 1
PD-L1: Programmed death 1 ligand
PgR: Progesterone receptor
RCB: Residual cancer burden
RFS: Recurrence-free survival
RT: Room temperature
TIL: Tumor-infiltrating lymphocyte
TNBC: Triple-negative breast cancer
