# Supplementary figures and images for: Prognostic significance of tumor-infiltrating CD8+ and FOXP3+ lymphocytes in residual tumors and alterations in these parameters after neoadjuvant chemotherapy in triple-negative breast cancer: a retrospective multicenter study
Source: Breast Cancer Res. 2015 Sep 4;17(1):124. doi: 10.1186/s13058-015-0632-x (PMC4560879; doi:10.1186/s13058-015-0632-x)

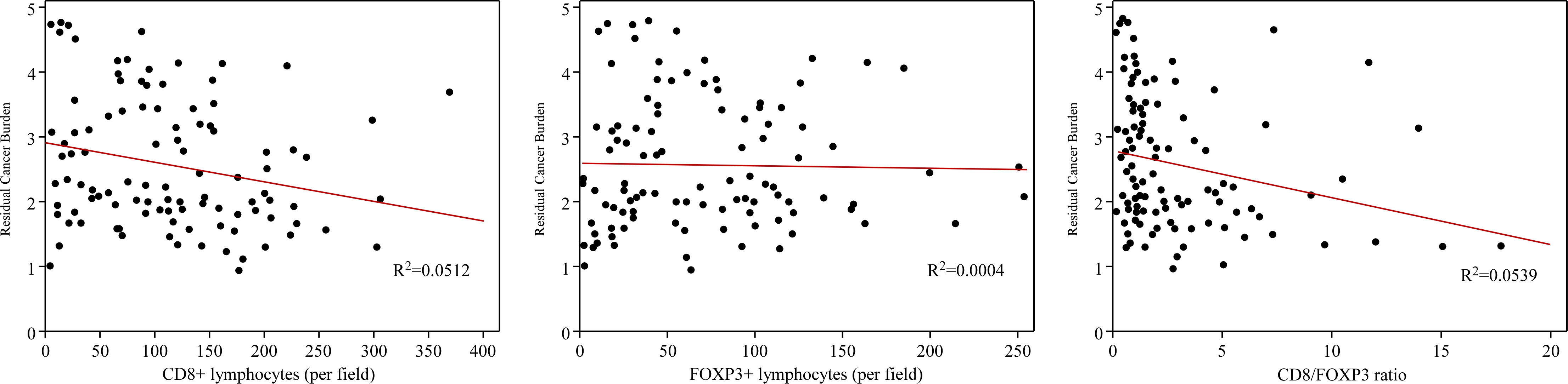

Supplement: Additional file 1: — The correlation diagrams of CD8 + TIL, FOXP3 + TIL, and CD8/FOXP3 ratio with residual cancer burden (RCB) score of each tumor. The weak inverse relations were detected between RCB scores and CD8+ TIL (A) or CD8/FOXP3 ratio (C), but this trend was not observed between RCB scores and FOXP3+ TIL (B). FOXP3 forkhead box protein 3, TIL tumor-infiltrating lymphocyte. (JPEG 599 kb) [file 13058_2015_632_MOESM1_ESM.jpg]

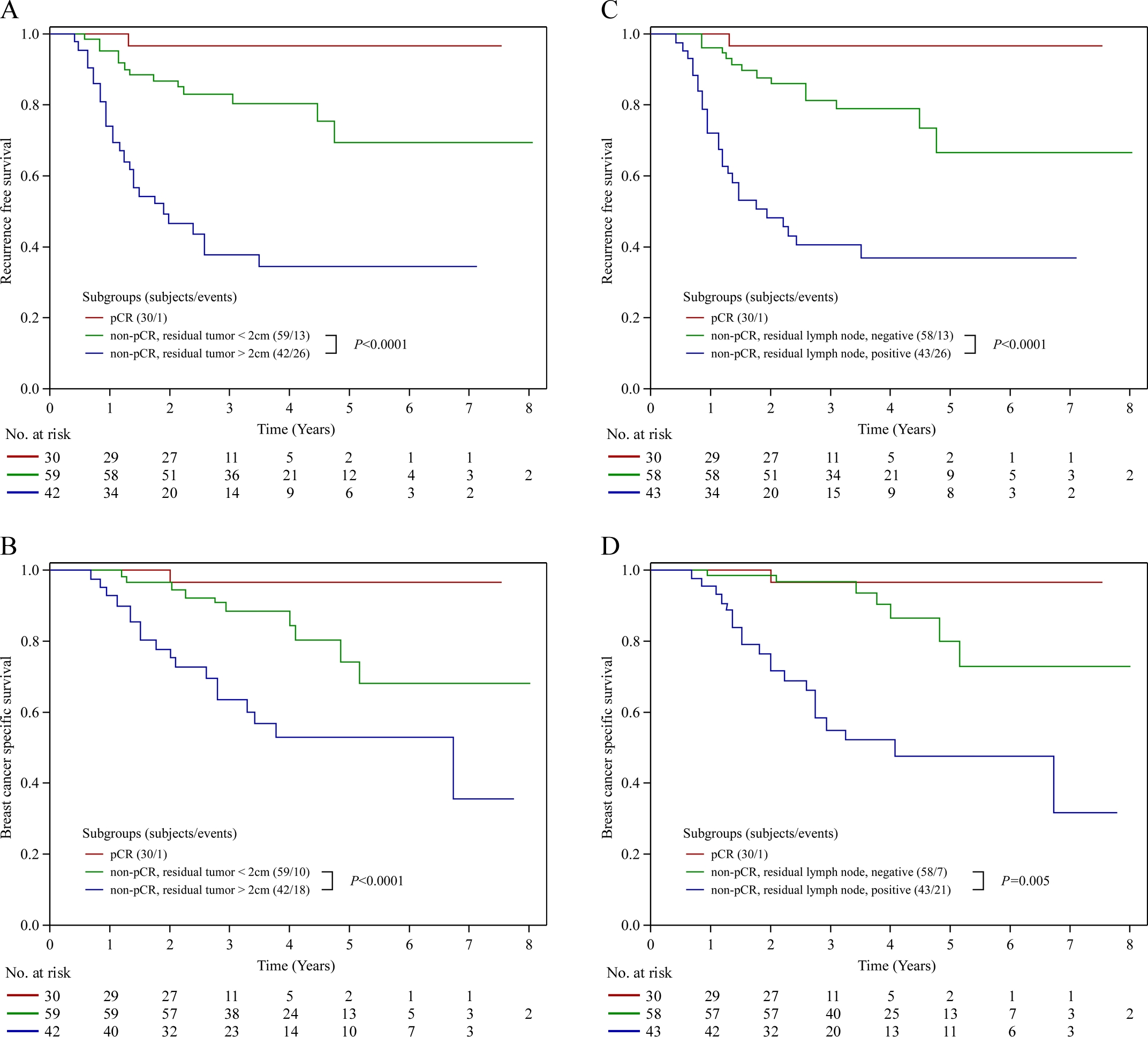

Supplement: Additional file 2: — Recurrence-free survival (RFS) and breast cancer-specific survival (BCSS) in patients with different residual tumor size and lymph node status. Estimated Kaplan-Meier curves of RFS (A) and BCSS (B) in patients with residual tumors of more than 2 cm or not more than 2 cm and those of RFS (C) and BCSS (D) in patients with metastatic lymph node-positive or -negative tumors. (JPEG 610 kb) [file 13058_2015_632_MOESM2_ESM.jpg]

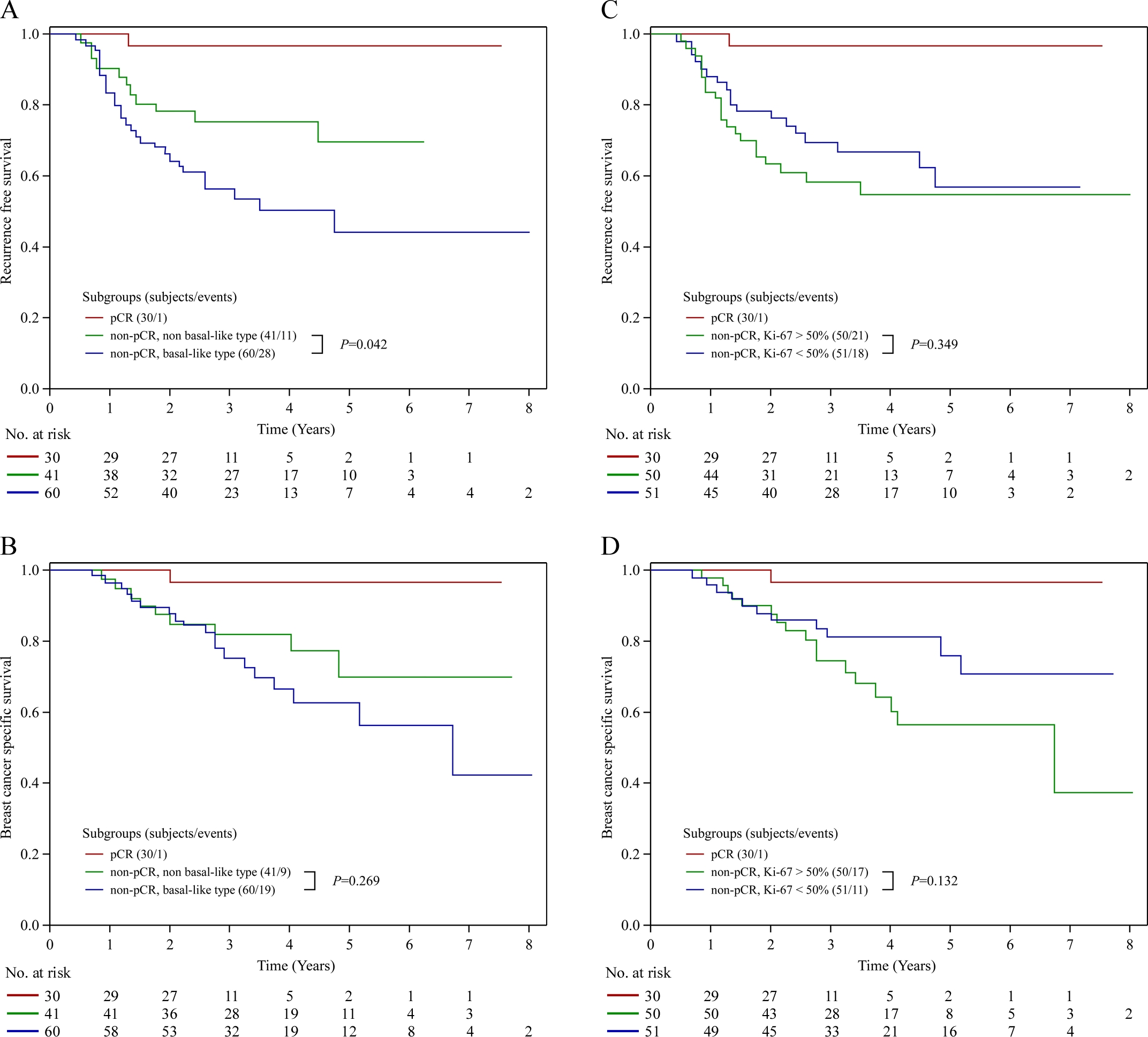

Supplement: Additional file 3: — Recurrence-free survival (RFS) and breast cancer-specific survival (BCSS) in patients with different status of basal-like feature and Ki-67 labeling index (LI). Estimated Kaplan-Meier curves of RFS (A) and BCSS (B) in patients with basal-like type or non-basal-like type and those of RFS (C) and BCSS (D) in patients with high or low Ki-67 LI. (JPEG 590 kb) [file 13058_2015_632_MOESM3_ESM.jpg]

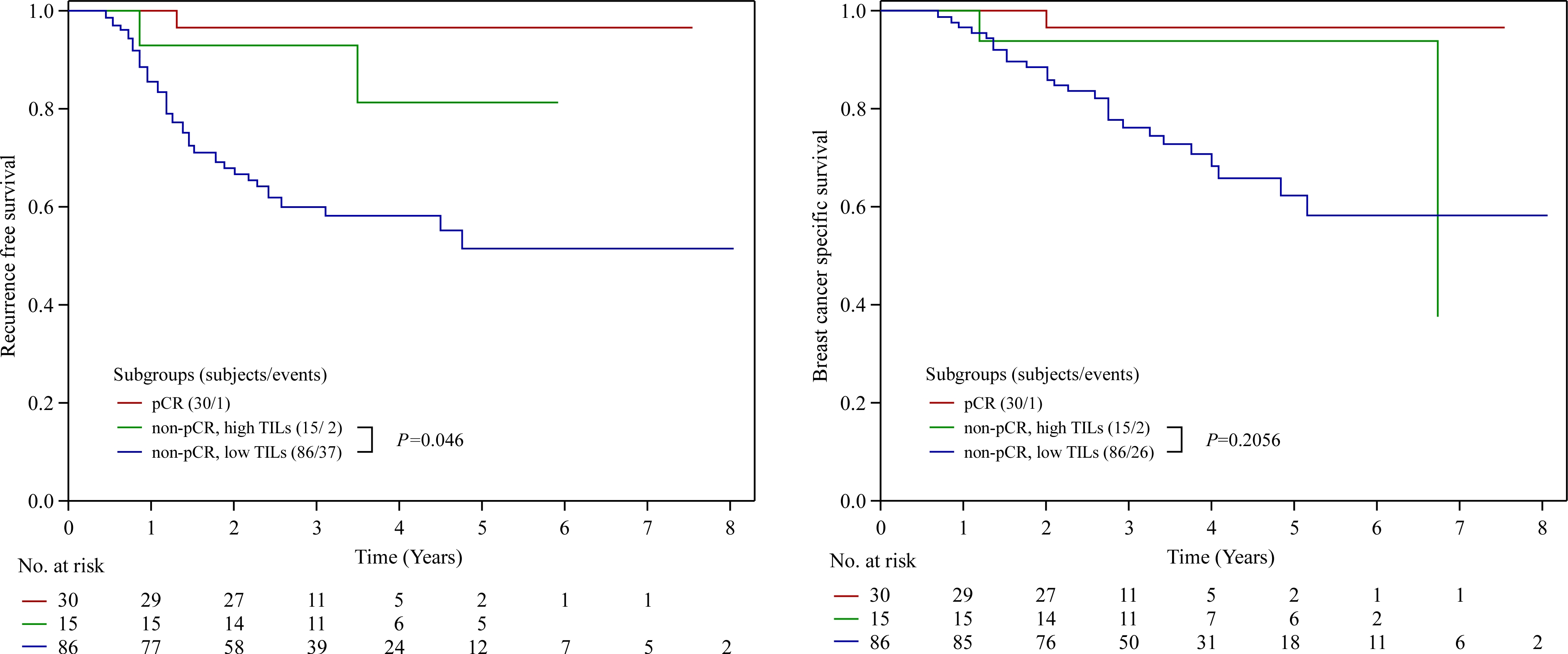

Supplement: Additional file 4: — Recurrence-free survival (RFS) and breast cancer-specific survival (BCSS) in patients with different status of tumor-infiltrating lymphocytes (TILs). Estimated Kaplan-Meier curves of RFS (A) and BCSS (B) in patients with high or low TILs. (JPEG 550 kb) [file 13058_2015_632_MOESM4_ESM.jpg]
